# Do Repeated Respiratory Tract Infections Predispose to Amoebic Meningoencephalitis Caused by Free-Living Amoebae?

**DOI:** 10.7759/cureus.66417

**Published:** 2024-08-08

**Authors:** Venkataramana Kandi

**Affiliations:** 1 Clinical Microbiology, Prathima Institute of Medical Sciences, Karimnagar, IND

**Keywords:** upper respiratory tract, immunocompromised, immunocompetent, opportunistic organisms, amoebic meningoencephalitis, free-living amoebae

## Abstract

Recent reports of fatal meningoencephalitis from Kerala, South India, are creating ripples among medical and health administrations. Free-living amoebae (FLA) have been attributed to these infections. Despite their free-living nature, these amoebae have been recognized as potential opportunistic organisms that occasionally cause infections in immunocompetent and immunocompromised individuals. The pathophysiology, epidemiology, and clinical course of diseases caused by FLA are not completely understood. Exposure to humans and animals is imminent given their presence in the air, water, and soil. Human infections, although infrequent, can lead to severe morbidity and mortality as evidenced by the recently emerging reports from Kerala, South India. Since these infections were noticed among children, it is unlikely that they were immunocompromised. Therefore, in this editorial, we speculate on the possibility of frequent upper respiratory tract viral infections as predisposing factors for infection with FLA.

## Editorial

Fatal primary meningoencephalitis caused by free-living amoebae (FLA) jolted the healthcare administration in Kerala, South India [1]. Three deaths in the past two months, all involving children aged less than 15 years, come as a surprise given the fact that these amoebae are non-pathogenic by nature. FLA are a group of unicellular protozoan parasites known for their survival in the environment feeding bacteria, fungi, and algae. These include *Acanthamoeba *species (spp.), *Naegleria *(*N*) *fowleri*, *Balamuthia mandrillaris*,* *and *Sappinia diploidea*. The latter two are frequently associated with animal infections and the former two cause opportunistic infections in humans. *Acanthamoeba *spp. causes granulomatous amoebic encephalitis (GAE) and amoebic keratitis in humans. It occurs in two morphological forms, including the trophozoite and the cyst. The trophozoite is the feeding and replicating form, whereas the cyst is a dormant form that allows the parasite to survive harsh environments. Human infections generally precede exposure to trophozoite forms that are accidentally inhaled by exposure to soil and fresh waters. The amoebae travel through the nasal passage and gain access to the brain by traveling through the sinuses, thereby causing GAE. GAE is a chronic condition, where infected patients present with symptoms of flu-like illness, fever, fatigue, nausea, vomiting, headache, and neck stiffness. Complications of GAE include the dissemination of the parasite, coma, and death. Radiological imaging and microscopic examination of the brain and cerebrospinal fluid (CSF) are used to identify the parasite and diagnose the infection. Other methods, including culture and antigen detection from appropriate patient specimens, can be used for laboratory diagnosis. There is no specific anti-parasitic drug to treat GAE caused by *Acanthamoeba *spp. However, some antibiotics and antifungal agents like amphotericin B and miltefosine, an experimental drug, were found effective against GAE. 

*N*. *fowleri *occurs in three morphological forms: the trophozoite, the cyst, and the intermediary flagellate. Most human infections occur due to the penetration of the trophozoite forms into the nasal mucosa while swimming in fresh water and on exposure to contaminated moist soil. The trophozoites travel through the optic nerve and reach the brain, causing primary amoebic meningoencephalitis (PAM). PAE is an acute infection wherein the patients develop symptoms like headache and neck stiffness three to six days after the exposure. After one or two weeks, patients develop complications like seizures and coma that may result in death. Diagnosis and treatment are like those for Acanthamoeba infection. A delay in the treatment could almost certainly result in the death of the patients [2].

The predisposing factors for GAE include patients suffering from acquired immunodeficiency syndrome (AIDS), cancer, hematological disorders, diabetes mellitus, liver cirrhosis, and organ transplant patients [3]. FLA causes infections in immunocompetent and immunocompromised individuals following swimming in fresh waters. The differential diagnosis of FLA infections is complex and involves bacterial, fungal, and other parasitic aetiologies [4].

**Figure 1:**
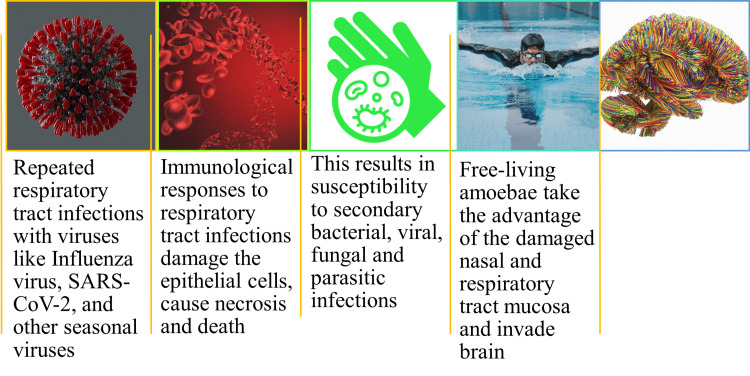
Probable mechanism for infection with free-living amoebae Image credit: Venkataramana Kandi SARS-CoV-2: severe acute respiratory syndrome coronavirus-2

The upper respiratory tract (URT) includes the nose, sinuses, throat/pharynx, and larynx. Seasonal viral infections caused by the influenza virus and respiratory tract infections (RTIs) caused by other viruses, including the severe acute respiratory syndrome coronavirus-2 (SARS-CoV-2), may result in the inflammation of the URT. Further, these infections induce oxidative stress, and cell/tissue injury, thereby predisposing such individuals to infections by FLA [5]. Figure 1 depicts the probable mechanism for infection with FLA.

Early identification of the cases is a prerequisite to prevent the death of the infected patients. Despite this, it is exceedingly difficult to save the patients who develop meningoencephalitis. No specific therapeutic drugs are available to treat infections caused by FLA. Repurposed drugs, including antifungal agents like amphotericin B, fluconazole, and miconazole, could be useful in treating patients. Antibiotics like paromomycin, polymyxin B, and sulfadiazine could also prove beneficial in treating *Acanthamoeba *infections. Given their ubiquitous nature, it is difficult to eliminate the parasites from the environment. However, water treatment with chlorination may prevent infections during swimming.

Measures including active surveillance of the FLA in the environment (soil and water) and animals could be instrumental in predicting, preventing, and controlling the disease spread. Further extensive animal and human studies are essential to improve understanding of the pathophysiology of diseases caused by FLA, especially related to predisposing factors like age, sex, co-morbidities, and other virulence determinants. This could contribute to developing improved therapeutic interventions and preventive and control measures.
